# NAFLD-associated immune remodeling in colorectal cancer liver metastasis: mechanisms and implications for immunotherapy

**DOI:** 10.3389/fimmu.2026.1863647

**Published:** 2026-06-29

**Authors:** Hui Wang, Guoqing Gu

**Affiliations:** 1 Health Management Center, Geriatric Hospital of Nanjing Medical University, Nanjing, China; 2 Gastroenterology Department, Geriatric Hospital of Nanjing Medical University, Nanjing, China

**Keywords:** CD8+ T cell exhaustion, colorectal cancer liver metastasis, immunosuppressive network, immunotherapy, myeloid-derived suppressor cells, non-alcoholic fatty liver disease, regulatory T cells

## Abstract

Non-alcoholic fatty liver disease (NAFLD) affects over 30% of the global adult population and has been increasingly recognized as an adverse prognostic factor for colorectal cancer (CRC) liver metastasis (CRLM). However, the underlying immune mechanisms remain incompletely understood. This review provides a comprehensive synthesis of how NAFLD-induced lipotoxicity reshapes the hepatic immune microenvironment to create a “fertile soil” for CRC metastatic colonization. We delineate a hierarchical immunosuppressive network involving: (1) lipotoxic apoptosis of CD4+ T cells, depleting the helper T cell pool; (2) expansion and functional reinforcement of regulatory T cells (Tregs) through multiple mechanisms (IL-10/TGF-β/IL-35, CD25-mediated IL-2 competition, CTLA-4 engagement, and granzyme B/perforin production); (3) M2 polarization of tumor-associated macrophages; and (4) recruitment of myeloid-derived suppressor cells (MDSCs) via the CXCL5/CXCR2 axis, which suppress CD8+ T cell function. These processes collectively promote CD8+ T cell exhaustion (marked by PD-1, LAG-3, TIM-3 upregulation) and spatial mismatch with metastatic foci. Importantly, we discuss the emerging evidence linking NAFLD-driven immune dysregulation to reduced responsiveness to immune checkpoint inhibitors, while acknowledging the current limitations in direct clinical validation. By integrating mechanistic insights with therapeutic perspectives, this review offers new targets for risk stratification and combination immunotherapy in the growing NAFLD population. Non-alcoholic fatty liver disease.

## 1 Introduction

Non-alcoholic fatty liver disease (NAFLD) represents a significant worldwide health burden, with over 30% of adults affected and numbers continuing to climb, driven by rising obesity and metabolic syndrome. NAFLD has now become a leading reason for end-stage liver disease, surpassing viral hepatitis (1). Colorectal cancer (CRC) ranks as the third most common cancer around the world, and the liver is the primary site where it spreads distantly. Roughly one-quarter of CRC patients present with liver metastases at the time of diagnosis, and another quarter to half will develop such metastases later in their illness (2).

Blood-based biomarkers have emerged as promising tools for early CRC detection because they are minimally invasive and can be measured from plasma or serum samples. A recent systematic review of blood-based biomarkers for early CRC detection summarized 142 studies across DNA, RNA, and protein markers and reported several promising candidates, while emphasizing that most require further validation because of small cohorts, selection bias, and limited cost-effectiveness data (3).

Epidemiological data reveal a tight connection between NAFLD and CRC liver spread. CRC patients who also have NAFLD show a notably greater frequency of liver metastases, quicker growth of those metastases, and a worse outlook (4). This link has spurred intense investigation into how the “liver microenvironment” affects the metastatic process. The classic “seed and soil” hypothesis tells us that successful tumor cell colonization (the seeds) relies heavily on the nature of the target organ’s microenvironment (the soil). Changes in the liver microenvironment brought on by NAFLD, especially immune-related alterations, may convert the liver into a more welcoming “soil” for tumor cells to take root (5).

The liver is unique in combining metabolic and immune roles. As NAFLD moves from simple steatosis to steatohepatitis (NASH), the liver’s immune microenvironment undergoes extensive remodeling, affecting both innate and adaptive immune cells. Recently, adaptive immune imbalance—particularly problems with regulatory T cells (Tregs) and effector T cells—has drawn growing interest in relation to NAFLD-associated liver cancer. Nevertheless, exactly how these immune changes influence CRC liver metastasis remains poorly understood.

Of note, the nomenclature of fatty liver disease has evolved from NAFLD/NASH to MASLD/MASH. In this review, we primarily use NAFLD/NASH to maintain consistency with older literature, but the terms are used interchangeably.

This review offers a systematic look, from an adaptive immunity angle, at how the hepatic immune microenvironment is reshaped in NAFLD. We concentrate on how Treg expansion and heightened function, together with CD8+ T cell exhaustion and death, build an immunosuppressive network that helps CRC cells evade immunity and establish metastases. We also address the translational meaning of these findings for prognosis and immunotherapy planning.

## 2 NAFLD-associated lipotoxicity

The Initial Spark for Hepatic Immune Remodeling.

At the heart of NAFLD’s pathophysiology lies lipotoxicity, stemming from disordered lipid metabolism in the liver. An overload of free fatty acids (FFAs) and their breakdown products not only injures hepatocytes directly but also starts and sustains changes in the hepatic immune microenvironment through various pathways.

Lipotoxic metabolites activate the innate immune system via damage-associated molecular patterns (DAMPs). Saturated fatty acids such as palmitic acid bind to Toll-like receptor 4 (TLR4), pushing hepatocytes and Kupffer cells to release pro-inflammatory cytokines. At the same time, cholesterol crystals set off the NLRP3 inflammasome, causing maturation and release of IL-1β and IL-18. These inflammatory signals recruit peripheral immune cells into the liver and also directly alter the characteristics and actions of local immune cells (6). After liver injury, Kupffer cells quickly react to DAMPs by sending out chemokines like CCL2 and CXCL10, which draw circulating monocytes into the liver, where they turn into monocyte-derived macrophages (MoMFs). Through CXCR3, CXCL10 specifically lures cytotoxic T lymphocytes and natural killer (NK) cells to inflamed liver sites, adding to hepatocyte death and inflammatory reactions (7). Guided by local signals, the recruited macrophages shift toward either an M1 (pro-inflammatory) or M2 (immunosuppressive/tissue repair) state (8). Recent single-cell sequencing work has uncovered new subpopulations, including scar-associated macrophages (SAMs) and lipid-associated macrophages (LAMs), which have specialized functions in fibrosis and metabolic dysfunction-associated fatty liver disease (6).

Transcriptional dysregulation also plays an important role in MASLD progression. Recent work identified KLF10 as a protective regulator in MASH, showing that hepatic KLF10 is reduced in MASH patients and obese mouse models, and that hepatocyte KLF10 overexpression protects against Western diet-induced steatohepatitis and hypercholesterolemia through HNF4α-mediated regulation of lipid, cholesterol, and bile acid metabolic pathways (9).

More critically, the lipotoxic setting directly influences adaptive immune cells. FFAs can enter CD4+ T cells, causing mitochondrial problems and a burst of reactive oxygen species (ROS), which triggers cell death. This process selectively reduces the CD4+ T cell population, weakening the helper support that these cells normally give to CD8+ T cells. Simultaneously, lipid metabolites such as oxysterols and certain bile acid derivatives encourage Treg differentiation and stability. This differential regulation of immune cells provides the molecular underpinning for hepatic immune imbalance in NAFLD (10).

## 3 How adaptive immune imbalance promotes CRC liver metastasis in NAFLD

The lipotoxic environment in NAFLD reprograms adaptive immunity in the liver through several routes, most notably by expanding Tregs and boosting their suppressive power, while also causing effector T cells to become exhausted or die. Together, these changes create an immunosuppressive network that helps CRC cells evade attack and settle in the liver.

### 3.1 Overview: a stepwise cascade of adaptive immune dysregulation

First, the lipotoxic environment directly harms CD4+ T cells (the starting event). Next, this leads to an increase in Treg numbers and stronger suppressive activity (the master organizers of immunosuppression). Finally, CD8+ T cells become exhausted (effector failure), completing a self-perpetuating loop.

### 3.2 Direct lipotoxic damage to T cells

The first step in adaptive immune imbalance is the lipotoxic death of CD4+ T cells. In NAFLD, FFAs that build up in the liver (e.g., linoleic acid, palmitic acid) can be taken up by CD4+ T cells. Too much fatty acid uptake disrupts the mitochondrial electron transport chain, lowers membrane potential, and triggers a ROS burst, eventually causing apoptosis. This reduces the number of CD4+ T cells in the liver and impairs the help they provide for CD8+ T cell growth, survival, and function (11, 12).

Metabolic reprogramming of T cells makes the problem worse. The lipotoxic environment pushes T cells that enter the liver to change their metabolism, shifting from oxidative phosphorylation toward more glycolysis. But this adaptation is often incomplete, leaving T cells in a “metabolic crisis”—not enough energy and a buildup of inhibitory products—which prepares them for later exhaustion.

### 3.3 Treg expansion, recruitment, and stronger suppressive function

Tregs are central to adaptive immune imbalance. In NAFLD/MASH livers, Treg numbers go up significantly, and their ability to suppress immunity becomes much stronger.

Several factors drive Treg enrichment in the fatty liver. Oxysterols directly encourage Treg differentiation. Products of gut microbes, such as short-chain fatty acids and secondary bile acids (e.g., isoallo-LCA), boost Treg growth and stability by activating RORγt or raising Foxp3 levels. Higher amounts of TGF-β and retinoic acid in the liver also favor Treg formation. In addition, hepatic stellate cells and Kupffer cells bring in peripheral Tregs by releasing chemokines like CCL17 and CCL22 (13).

**Figure 1:**
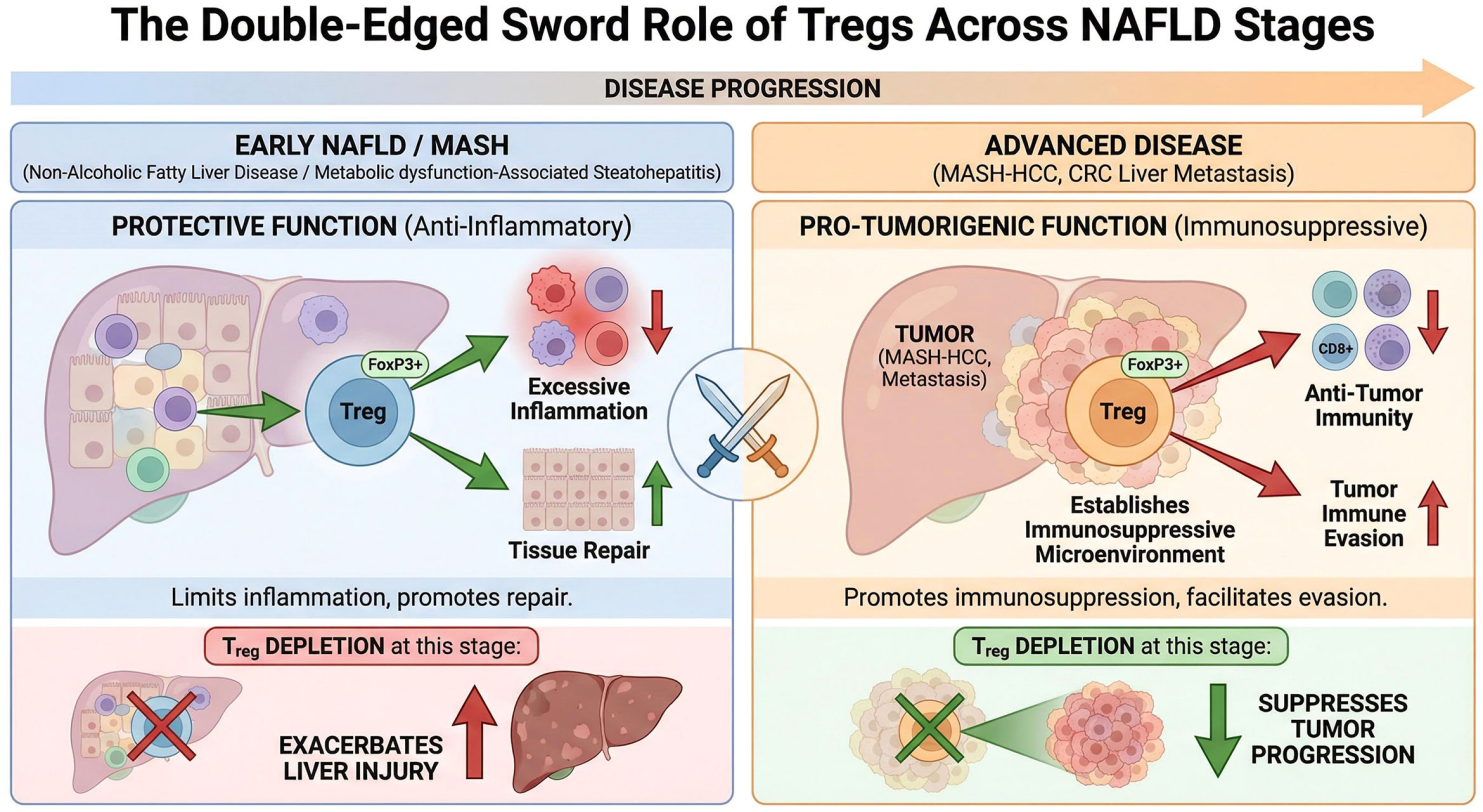
NAFLD-associated lipotoxicity (free fatty acids, cholesterol crystals) drives three parallel pathological processes: (1) lipotoxic apoptosis of CD4+ T cells, depleting the helper T cell pool; (2) expansion and functional enhancement of regulatory T cells (Tregs) via multiple mechanisms (IL-10/TGF-β/IL-35 secretion, CD25-mediated IL-2 competition, CTLA-4 engagement, and granzyme B/perforin production); and (3) M2 polarization of macrophages. These processes collectively establish an immunosuppressive network that promotes CD8+ T cell exhaustion (characterized by PD-1, LAG-3, and TIM-3 upregulation and loss of cytotoxic molecules) and spatial mismatch with metastatic foci, ultimately facilitating immune evasion and accelerated growth of colorectal cancer liver metastases. Myeloid-derived suppressor cells (MDSCs) recruited via the CXCL5/CXCR2 axis further amplify immunosuppression.

The stronger immunosuppressive action of Tregs shows up in multiple ways. First, Tregs directly curb CD8+ T cell growth and killing ability by putting out inhibitory cytokines including IL-10, TGF-β, and IL-35 (14, 15). Second, Tregs carry large amounts of CD25 (the IL-2 receptor α chain), competing with effector T cells for IL-2 and causing effector T cells to die (16). Third, Tregs use surface CTLA-4 to bind CD80/CD86 on antigen-presenting cells, blocking their antigen-presenting role and thus indirectly weakening effector T cell activation (17). Fourth, Tregs can make granzyme B and perforin to directly kill effector T cells (18). The overall immunosuppressive network and its downstream effects on CD8 T cell function and tumor progression are summarized **Figure 1**.

The changing, “double-edged sword” nature of Tregs deserves careful thought. In early NAFLD (simple steatosis), Tregs may guard the liver by holding back too much inflammation. But as the disease moves to MASH and later to cancer, Tregs instead push immunosuppression and fibrosis, eventually helping tumors evade immunity. This variation over time and space has major implications for clinical treatment strategies.

In the early stage (simple steatosis or MASH), Tregs play a vital protective part in keeping the liver’s immune-metabolic balance. Studies have shown that in established MASH mouse models, Treg buildup comes with higher TIGIT and IL-10 levels, greatly reducing hepatocyte death and fibrosis—suggesting Tregs are essential “protectors” of the liver at this point.

But as the disease advances to MASH-linked liver cancer, Tregs change into key drivers of the tumor-friendly environment. Wang and colleagues found that in a NASH-induced liver cancer model, Tregs directly aid cancer formation by setting up an immunosuppressive setting, and removing Tregs strongly slows tumor growth (19). This shift from “liver defender” to “tumor helper” clearly shows how dynamic Treg function can be.

**Figure 2:**
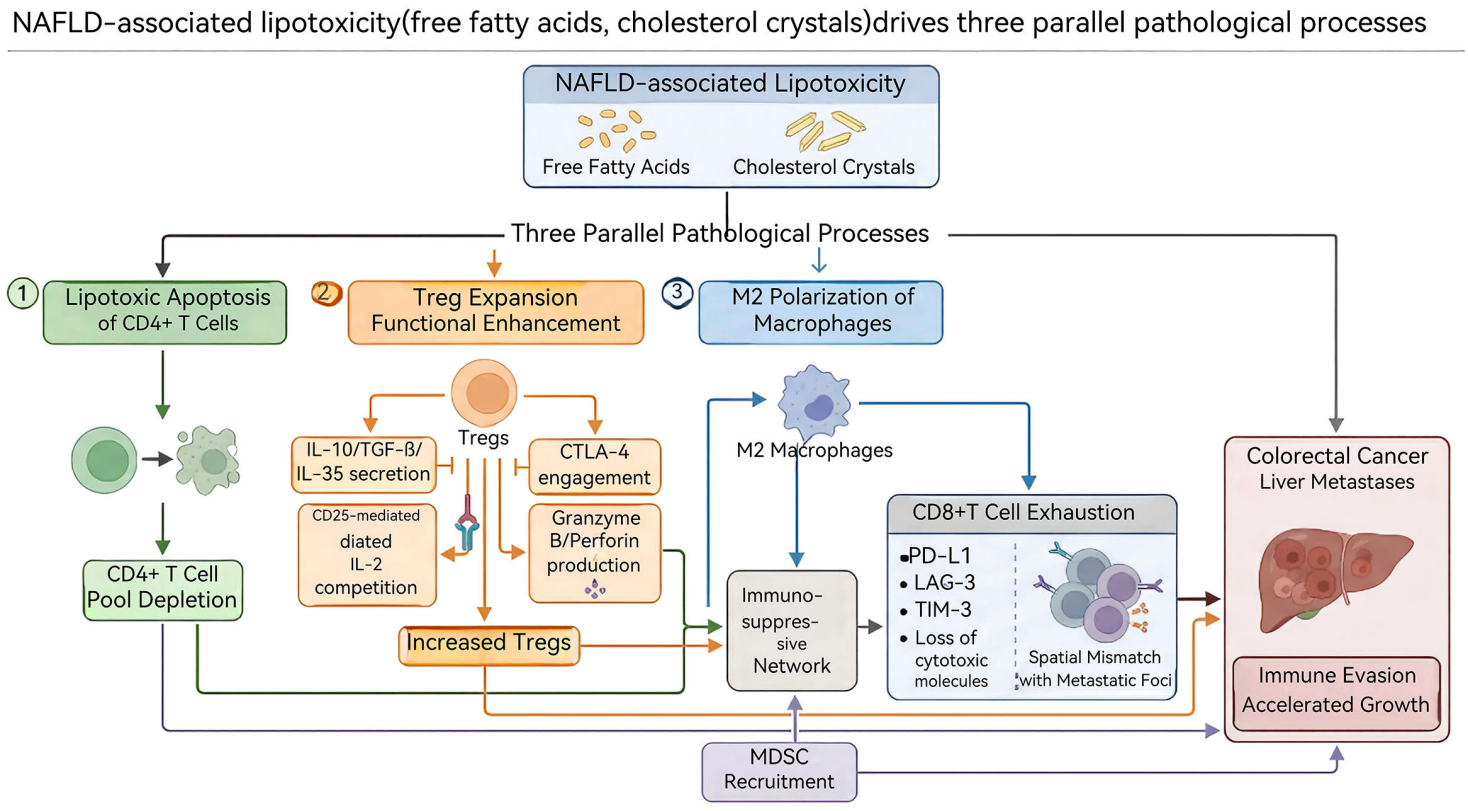
The double-edged sword role of Tregs across NAFLD stages. In early NAFLD (simple steatosis and MASH), Tregs exert protective functions by limiting excessive inflammation and promoting tissue repair. Treg depletion at these stages exacerbates liver injury. However, as disease progresses to MASH-associated HCC and CRC liver metastasis, Tregs are reshaped into pro-tumorigenic cells that establish an immunosuppressive microenvironment and facilitate tumor immune evasion. Treg depletion at advanced stages suppresses tumor progression. This spatiotemporal heterogeneity highlights the critical importance of timing when designing Treg-targeted therapies.

Thus, when designing treatments that target Tregs, getting the timing right is crucial. Getting rid of Tregs in the early stage might backfire, whereas going after Tregs or the pathways that create them (e.g., NETs) in the cancer stage could be an effective way to restore anti-tumor immunity. **Figure 2** illustrates this stage-dependent functional switch of Tregs across NA progression. This understanding sets the stage for later discussion of Treg-targeted clinical approaches.

### 3.4 CD8+ T cell exhaustion and loss of function

In NAFLD, CD8+ T cells show a clear exhaustion pattern. Both animal models and human samples confirm that CD8+ T cells entering MASH livers have high levels of exhaustion markers PD-1, LAG-3, and TIM-3 while keeping the tissue-resident marker CXCR6. These “tissue-resident exhausted CD8+ T cells” lose the capacity to produce IFN-γ, TNF-α, and granzymes, and cannot effectively clear tumor cells that arrive in the liver (20).

What drives CD8+ T cell exhaustion? Both ongoing antigen exposure and inhibitory signals play roles. In the lipotoxic setting, persistent inflammation and hepatocyte injury mean that antigens keep being presented; at the same time, inhibitory cytokines from Tregs and the larger immunosuppressive network push CD8+ T cells toward exhaustion. Pfister and colleagues showed that in a NASH-induced liver cancer mouse model, CD8+PD-1+ T cells in the liver were greatly increased, but these cells not only failed to control tumors—they actually worsened liver injury and fibrosis (21).

The loss of CD4+ T cell help makes CD8+ T cell exhaustion even worse. Because CD4+ T cells die in large numbers in the lipotoxic environment, CD8+ T cells cannot get enough “help” to keep long-term memory and killing power. This makes CD8+ T cells more likely to become exhausted when antigens keep appearing (22).

### 3.5 Treg interactions with other immune cells: forming the immunosuppressive network

Tregs do not work alone; they talk to many other immune cell types to build a complex immunosuppressive web.

The Treg-macrophage connection is a key hub. Tregs push macrophages toward the M2 type (tumor-associated macrophages, TAMs) by releasing factors like IL-13 and TGF-β; in response, M2 macrophages call in more Tregs using chemokines such as CCL2, CCL5, and CCL17, creating a positive feedback loop. Each M2 macrophage subtype (M2a, M2c, M2d) makes several chemokines; the M2a subtype has high levels of CCL17/18/22, which bring Tregs into the tumor microenvironment by binding to CCR4 on Treg surfaces. The M2c and M2d subtypes further boost local immunosuppression by putting out TGF-β and IL-10. This positive feedback between Tregs and M2 macrophages is a core part of the tumor immunosuppressive network and is especially strong in NAFLD—the lipotoxic environment both increases Tregs and drives M2 polarization, and their combined effects help CRC liver metastases evade immunity (23). Ohashi and coworkers showed that in NAFLD, tumor-associated macrophages produce IL-1β through NLRC4 inflammasome activation, encouraging M2 polarization and VEGF production, thus speeding up liver metastatic tumor growth (24).

Myeloid-derived suppressor cells (MDSCs) constitute a heterogeneous population of immature myeloid cells with potent immunosuppressive activity (25). Two major subsets have been characterized: monocytic MDSCs (M-MDSCs) and granulocytic/polymorphonuclear MDSCs (G-MDSCs/PMN-MDSCs) (26). M-MDSCs primarily suppress T cells through inducible nitric oxide synthase (iNOS) and arginase-1 (Arg-1), whereas G-MDSCs predominantly produce reactive oxygen species (ROS) (27). Importantly, the phenotypic markers for MDSCs differ substantially between mice and humans: murine MDSCs are typically defined as CD11b+Gr-1+, whereas human MDSCs are characterized by CD33+CD11b+HLA-DR-/low (26). This species difference must be considered when extrapolating findings from preclinical models to human disease. Moreover, MDSCs exhibit remarkable functional plasticity, with their suppressive capacity modulated by hypoxia, cytokines, and metabolic cues within the tumor microenvironment (28).

Recruitment and activation of MDSCs add even more immunosuppression. A study by Yang et al. suggested that NAFLD may induce Kupffer cells to secrete the chemokine CXCL5, which draws CXCR2+ MDSCs into the liver via the CXCL5/CXCR2 axis. These MDSCs block T cell function in several ways, including using up arginine in the environment, making ROS, and causing Treg expansion, thereby promoting liver metastatic tumor growth (29). While this study provides important mechanistic insights, further validation in CRC-specific cohorts and independent datasets is warranted.

The Treg–hepatic stellate cell (HSC) interaction is also worth noting. Activated HSCs not only drive fibrosis but also help Treg differentiation by releasing TGF-β and retinoic acid; in turn, Tregs keep HSCs activated by secreting factors like IL-10. This connection tightly links fibrosis with immunosuppression (30).

The teamwork between MDSCs and Tregs creates an “immunosuppressive double backup.” MDSCs can encourage Treg induction and growth through several routes, including making TGF-β, using up arginine, and expressing CD40; Tregs, in response, make a more welcoming environment for MDSCs by holding back effector T cell function. Their cooperation builds a strong barrier against immunity.

### 3.6 Additional components of the hepatic metastatic niche

Beyond the core immune cell populations discussed above, several other components of the tumor microenvironment contribute to the permissive “soil” for CRC liver metastasis in NAFLD.

Cancer-associated fibroblasts (CAFs): CAFs are activated fibroblasts that promote extracellular matrix remodeling, angiogenesis, and immunosuppression. Emerging evidence indicates that CAFs interact with Tregs and MDSCs through chemokine secretion (e.g., CXCL12, CCL2) and metabolic crosstalk, thereby enhancing the immunosuppressive network (31).

Gut-liver axis: The gut microbiota and its metabolites (short-chain fatty acids, secondary bile acids, lipopolysaccharide) profoundly influence hepatic immunity. Dysbiosis associated with NAFLD can promote intestinal permeability, leading to translocation of bacterial products into the portal circulation, which in turn activates hepatic innate immune cells and exacerbates inflammation (32).

Fibrosis: As NAFLD progresses to NASH, hepatic fibrosis develops and creates a physical barrier that may impede effector T cell infiltration while providing a scaffold for immunosuppressive cell accumulation. Fibrosis also alters mechanical signaling and metabolic gradients within the metastatic niche (33).

Metabolic reprogramming: The lipotoxic environment drives metabolic competition between tumor cells, immune cells, and stromal cells. Increased lipid availability can shift T cell metabolism toward fatty acid oxidation, impairing glycolytic flux required for effector function. Understanding these metabolic interactions may reveal novel therapeutic targets (10).

### 3.7 Poor positioning of CD8+ T cells relative to metastatic tumors in fatty livers

Recent research has revealed another key aspect of adaptive immune imbalance—a spatial disconnect between immune cells and metastatic deposits. Yehezkel and colleagues, using single-cell RNA sequencing and immunohistochemistry, discovered that although total CD8+ T and NK cell numbers rose in MASH livers, their entry into metastatic lesions was much lower, with most cells staying in the liver tissue around the metastases. Also, these “lost” CD8+ T cells had much less of the cytotoxic markers granzyme B and perforin (5).

This finding carries important clinical weight: more immune cells does not mean better function; what matters is whether they can actually get to the tumor site and carry out their killing job. Both the abnormal positioning and the poor function of CD8+ T cells in MASH livers help explain why anti-tumor immune surveillance fails.

## 4 Clinical translation: from mechanisms to prognosis

### 4.1 How NAFLD affects prognosis in CRC patients with liver metastasis

Clinical studies consistently report that CRC patients who also have NAFLD face a higher chance of liver metastasis and a worse outcome. Multicenter cohort studies have identified NAFLD as an independent risk factor for CRC liver metastasis; compared to patients without NAFLD, those with NAFLD and liver metastases have clearly shorter overall survival and disease-free survival (34).

The immune reasons for this difference in prognosis are slowly becoming clear. Yang et al. confirmed in clinical work that infiltration of CXCR2+ MDSCs is much higher in liver metastases of patients with fatty liver, and the density of MDSC infiltration is linked to worse patient outcomes (29). Yehezkel et al. further showed that CD8+ T cell killing function in the liver of MASH patients is impaired and is tied to the risk of liver metastasis (5).

### 4.2 Impact on how well immunotherapy works

The effect of NAFLD on immunotherapy success is especially striking. Pfister and colleagues, in a meta-analysis of three randomized trials, found that the benefit from immunotherapy varies greatly by cause—patients with viral hepatitis-related liver cancer got significantly more benefit from immunotherapy than those with non-viral causes. In two separate HCC groups, anti-PD-1/PD-L1 treatment was linked to shorter overall survival in patients with NASH-related HCC, no longer survival (21).

The mechanisms underlying this phenomenon are being actively investigated. A study by Yang et al. suggested that in the NAFLD setting, CRC liver metastases may not respond to anti-PD-1 alone; however, when the CXCR1/2 inhibitor Reparixin was added, the combination showed efficacy in preclinical models (29). While this provides preliminary evidence that MDSC-driven immunosuppression could contribute to immunotherapy resistance in NAFLD, direct clinical validation in CRC patients remains limited. Additional studies are needed to confirm these findings and to explore other potential resistance mechanisms, such as Treg accumulation, metabolic dysfunction, and altered antigen presentation.

### 4.3 Possible therapeutic targets based on immune mechanisms

Based on the mechanistic studies above, several approaches that target adaptive immune imbalance look promising.

Going after Tregs is one important direction. In MASH-related liver cancer models, removing Tregs or blocking their function can restore anti-tumor immune responses. But because Tregs protect the liver in early NAFLD, when to intervene must be chosen carefully (19).

Targeting MDSCs has more direct potential for clinical use. Reparixin (a CXCR1/2 blocker) together with anti-PD-1 antibodies wiped out tumors in a mouse model of CRC liver metastasis in the NAFLD setting, and this approach has moved into human trials (29).

Reversing T cell exhaustion is another key strategy. Blocking several immune checkpoints at once (e.g., PD-1 plus LAG-3 or TIM-3) or adding metabolic interventions (e.g., improving the lipotoxic environment) might more effectively restore CD8+ T cell function (35, 36).

## 5 Summary and outlook

This review has systematically laid out how adaptive immune imbalance in the liver promotes colorectal cancer liver metastasis in the setting of NAFLD, focusing on the roles of Treg expansion and stronger function, CD8+ T cell exhaustion, and the creation of immunosuppressive networks (including MDSCs) that reshape the “lipotoxic soil.” These immune changes not only explain the clinically seen higher risk of CRC liver metastasis in NAFLD patients but also give a theoretical basis for prognosis assessment and treatment improvement.

Overcoming immune checkpoint resistance in NAFLD-associated CRLM: The emerging evidence linking NAFLD to reduced immunotherapy efficacy underscores the need for novel combination strategies. Preclinical studies suggest that targeting MDSCs (e.g., with CXCR1/2 inhibitors), modulating Treg activity with precise timing, or combining immune checkpoint blockade with metabolic interventions (e.g., improving lipotoxicity, targeting cholesterol metabolism) may restore anti-tumor immunity. However, these approaches require rigorous testing in CRC-specific models and ultimately in clinical trials stratified by NAFLD status.

Still, current research has several shortcomings. First, differences remain between animal models and human disease; high-fat diet-induced NAFLD models cannot fully capture the complexity of human NAFLD. Second, the double-edged role of Tregs at different disease stages (simple steatosis vs. MASH vs. cancer) means that treatment timing must be precise. Third, how adaptive immune changes interact with other immune cells (e.g., B cells, NK cells) in NAFLD still needs thorough study.

Future research should include: using single-cell sequencing and spatial transcriptomics to build high-detail maps of the hepatic immune microenvironment in NAFLD; deeply exploring how metabolism and immunity talk to each other; developing precise treatments that target specific immune cell subsets; and running immunotherapy trials that separate patients by disease cause.

In closing, a deeper grasp of NAFLD-related immune imbalance will give new targets and theoretical foundations for preventing CRC liver metastasis and creating combination immunotherapy strategies. In the era of precision medicine, adding liver disease cause to risk assessment and treatment choices for CRC patients with liver metastasis should help improve outcomes for this high-risk group.
